# Renal tissue-resident macrophages promote cystogenesis in early polycystic kidney disease

**DOI:** 10.1242/jcs.263992

**Published:** 2025-08-26

**Authors:** Rudolfo Karl, Arsila Palliyulla Kariat Ashraf, Maria Francesca Viola, Katharina Hopp, Elvira Mass, Dagmar Wachten

**Affiliations:** ^1^ Institute of Innate Immunity, Biophysical Imaging, Medical Faculty, University of Bonn, Bonn 53127, Germany; ^2^ Life and Medical Sciences (LIMES) Institute, Developmental Biology of the Immune System, University of Bonn, Bonn 53115, Germany; ^3^ Department of Medicine, Division of Renal Diseases and Hypertension, Polycystic Kidney Disease Program, University of Colorado Anschutz Medical Campus, Aurora, CO 80045, USA

**Keywords:** Cilia, PKD, Macrophages, Kidney

## Abstract

Autosomal-dominant polycystic kidney disease (ADPKD) is a ciliopathy characterized by mutations in *PKD1* or *PKD2*, which drive cystogenesis in renal epithelial cells. Immune cells, particularly macrophages, contribute to disease progression, yet their role remains incompletely understood. Here, we performed an in-depth analysis of renal macrophage ontogeny and phenotype and investigated their function in an ADPKD mouse model (*Pkd1*^RC/RC^) with adult onset and slow disease progression. We demonstrate that the numbers of tissue-resident macrophages were already increased before cyst formation. Using a flow cytometry screening panel, we further characterized the tissue-resident macrophage populations using surface markers and identified a novel marker that shows the potential to determine macrophage remodeling at different disease stages. To reveal the cellular interaction of tissue-resident macrophages and renal epithelial cells in further detail, we established a 3D co-culture system, demonstrating that tissue-resident macrophages from *Pkd1*^RC/RC^ mice, isolated at a stage before cysts were observed, already showed enhanced cystogenesis *in vitro*. These findings underscore the crucial role of tissue-resident macrophages in ADPKD and suggest targeting epithelial cell–macrophage interactions as a promising therapeutic avenue.

## Teaser

**Summary:** Tissue-resident macrophages promote cystogenesis of renal epithelial cells during polycystic kidney disease, making epithelial–macrophage interactions a prime target for treatment.

## INTRODUCTION

Autosomal-dominant polycystic kidney disease (ADPKD) is a ciliopathy caused predominantly by mutations in the *PKD1* or *PKD2* gene, encoding for polycystin 1 (PC1) and PC2, respectively (Chapin and Caplan, 2010). So far, treatment options are limited; ADPKD patients are dependent on dialysis, and there is no causal treatment other than kidney transplantation. The only FDA-approved drug, Tolvaptan, delays cyst formation in renal epithelial cells, without being a curative option. Thus, understanding the molecular mechanisms underlying cyst development and disease progression is key to make progress in unraveling the pathomechanisms underlying polycystic kidney disease (PKD) and developing novel treatment options.

PC1 and PC2 form a protein complex in primary cilia, which conducts Ca^2+^ in response to fluid flow. Primary cilia dysfunction in ADPKD disrupts PC1 and PC2 function and, thereby, Ca^2+^ signaling in primary cilia of renal epithelial cells. This has been proposed to relieve the inhibition of Ca^2+^-dependent adenylyl cyclase (AC) isoforms AC5 and AC6 in primary cilia, thereby increasing ciliary cAMP levels (Choi et al., 2011; Nachury and Mick, 2019). In fact, loss of either AC5 or AC6 ameliorates cyst progression (Rees et al., 2014; Wang et al., 2018). Furthermore, cAMP levels are elevated in renal cysts *in vivo* (Yamaguchi et al., 1997), and an increase of intracellular cAMP levels in renal epithelial cells *in vitro* leads to cyst formation (Grantham, 1996)*.* Furthermore, controlling the activity of specific phosphodiesterase (PDE) families (i.e. PDE4 long isoforms) regulates PKD severity (Omar et al., 2019; Ye et al., 2016). Finally, Tolvaptan antagonizes the vasopressin 2 receptor (V2R; also known as AVPR2), a G_αs_-protein coupled receptor, which causes an increase in cellular cAMP levels upon stimulation (Ma et al., 2017; Torres and Harris, 2014). Shedding light on the cAMP-dependent mechanisms underlying ADPKD, we have applied optogenetics to manipulate ciliary cAMP signaling and identified a novel cAMP signalosome in primary cilia of renal epithelial cells that drives cystogenesis (Hansen et al., 2022). It relies on the cAMP-dependent activation of protein kinase A (PKA) in the cilium, which phosphorylates the transcription factor cyclic AMP-responsive element binding-protein 1 (CREB1). In turn, activated ciliary phosphorylated (p)CREB1 drives a gene expression program that is distinct from the gene expression program evoked by cAMP in the cytoplasm, leading to cyst formation (Hansen et al., 2022).

Although primary cilia dysfunction in renal epithelial cells and cAMP signaling downstream underlies PKD development, other cells in the tissue environment contribute to disease progression. Here, immune cells, in particular macrophages, have been shown to play a key role. In both, *PKD1* and *PKD2* mouse models, macrophage numbers are increased, with the cells lining the cysts (Karihaloo et al., 2011). Treatment with clodronate to deplete all phagocytic cells or deletion of *Cx3cr1*, a chemokine receptor expressed on kidney-resident macrophages, in an ADPKD mouse model, reduces the cystic index and improved renal function (Karihaloo et al., 2011; Li et al., 2023). Similar results have been observed in a mouse model for autosomal recessive PKD (Swenson-Fields et al., 2013). Thus, there is a clear correlation between macrophage function and disease progression.

Macrophages are the largest immune cell population in the kidney under homeostatic conditions (Duffield, 2010) and can be distinguished according to a combination of their functional properties and their ontogeny. Renal tissue-resident macrophages originate from yolk sac erythro-myeloid progenitors (EMPs). EMPs give rise to circulating pre-macrophages which colonize the kidney as early as embryonic day (E)14.5 (Gomez Perdiguero et al., 2015; Mass et al., 2016). It has been proposed that long-lived macrophages with self-maintaining properties are largely EMP-derived with a small fraction of short-lived monocyte-derived macrophages (MdMs) contributing to the renal macrophage pool (He et al., 2024; Yashchenko et al., 2023). However, this dichotomy between macrophage origin and longevity is likely oversimplified given that the applied fate-mapping models used so-far have been applied to adult animals and do not exclude long-lived MdMs with self-maintaining properties. Therefore, the contribution of renal macrophages of distinct origins to PKD onset and progression remain incompletely understood.

Here, we use an orthologous mouse model of ADPKD1 (Arroyo et al., 2021; Hopp et al., 2012) in a C57BL/6JRcc background (*Pkd1^RC/RC^*) to model a slow disease onset, characterize macrophage abundance and phenotype during disease progression, and investigate their effect on cyst development in a 3D model system *in vitro*. Together, our data demonstrate that the remodeling of tissue-resident macrophages has already occurred before cyst formation, which promotes cystogenesis.

## RESULTS AND DISCUSSION

### Renal macrophage ontogeny and longevity

**Fig. 1.**
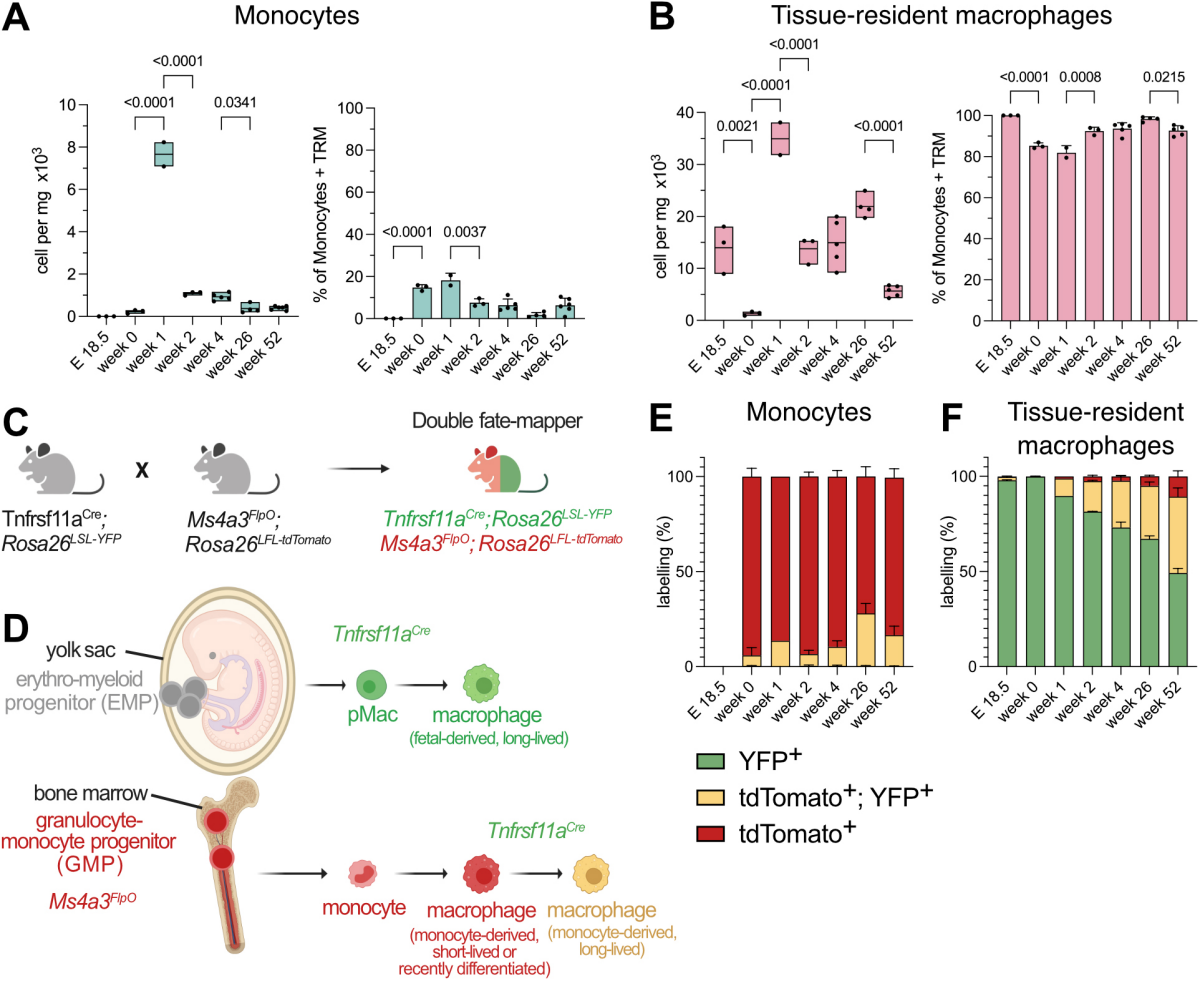
**Characterization of renal macrophage ontogeny and surface marker expression.** (A) Monocytes and (B) tissue-resident macrophages in the kidney, plotted as total cell count [box plots, where the box represents the range (minimum to maximum), and the mean is indicated] and as percentage of monocytes plus tissue-resident macrophages (mean±s.d.). *P*-values were calculated using a one-way ANOVA with Bonferroni correction. (C) Double fate-mapper model (Huang et al., 2025). (D) Schematic overview labelling macrophage ontogeny. Created in BioRender by Wachten, D., 2025. https://BioRender.com/mfe4ueq. This figure was sublicensed under CC-BY 4.0 terms. (E) Fate mapping of monocytes and (F) tissue-resident macrophages, depicted as fraction of the total renal macrophage population (set at 100%). Data are shown as mean±s.d. In A, B, E and F, *n*=3 (E18.5, week 0, 2), 2 (week 1), 4 (week 26), 5 (week 4), 6 (week 52).

To assess macrophage heterogeneity and macrophage and monocyte abundance in the kidney, we used flow cytometry. Based on surface marker expression, we distinguished between hematopoietic stem cell (HSC)-derived monocytes (Ly6C^+^, CD11c^−^) and tissue-resident macrophages (CD11b^+^, Ly6C^−^, F4/80^+^, CD11c^+^, MHCII^+^) (Fig. S1A). We analyzed the relative distribution of the different cell populations in wild-type mice during development and aging. Whereas the fetal kidney at E18.5 only contains tissue-resident macrophages, the contribution of monocytes increased in the first 3 weeks after birth but declined with age (Fig. 1A,B; Fig. S1B,C), suggesting an early influx of monocytes into the growing kidney and differentiation into MdMs.

To enable studying fetal-derived macrophages and MdMs in the same animal without the need of transplantation or tamoxifen injection, we created a novel mouse model that we have termed ‘double fate-mapper’ (Fig. 1C) (Huang et al., 2025). The double fate-mapper uses a combination of Cre and Flp recombinase activity, thereby labeling yolk sac-derived macrophages with yellow fluorescent protein (YFP), and monocytes and MdMs stemming from granulocyte-monocyte progenitors in the bone marrow with tdTomato (tdT, Fig. 1D) (Huang et al., 2025). Similar to what we observed in the peripheral blood (Huang et al., 2025), Ly6C^+^ monocytes in the kidney were all tdT^+^ with only a small fraction of tdT^+^YFP^+^ cells (Fig. 1E). During embryonic development, all CD11c^+^ tissue-resident macrophages were YFP^+^ and, therefore, yolk sac-derived (Fig. 1F). As expected from the data of wild-type animals (Fig. 1A), monocytes started to give rise to MdMs in the developing kidney. However, only a small fraction of monocytes (1–10%) were constantly recruited and differentiated into short-lived (tdT^+^) MdMs between 1 and 52 weeks after birth, whereas the majority of MdMs became long-lived (tdT^+^YFP^+^) and tissue resident (Fig. 1F). At 26–52 weeks of age, 50–65% of tissue-resident macrophages remained yolk sac derived (Fig. 1F). In summary, our data reveal that macrophage ontogeny during early postnatal development is highly dynamic, but that at 4 weeks of age, the kidney harbors mostly yolk-sac-derived macrophages. Only during aging, are macrophage populations are partially replaced by MdMs that take up tissue residency.

### Renal tissue-resident macrophages are increased in *Pkd1^RC/RC^* kidney at an early disease state

**Fig. 2.**
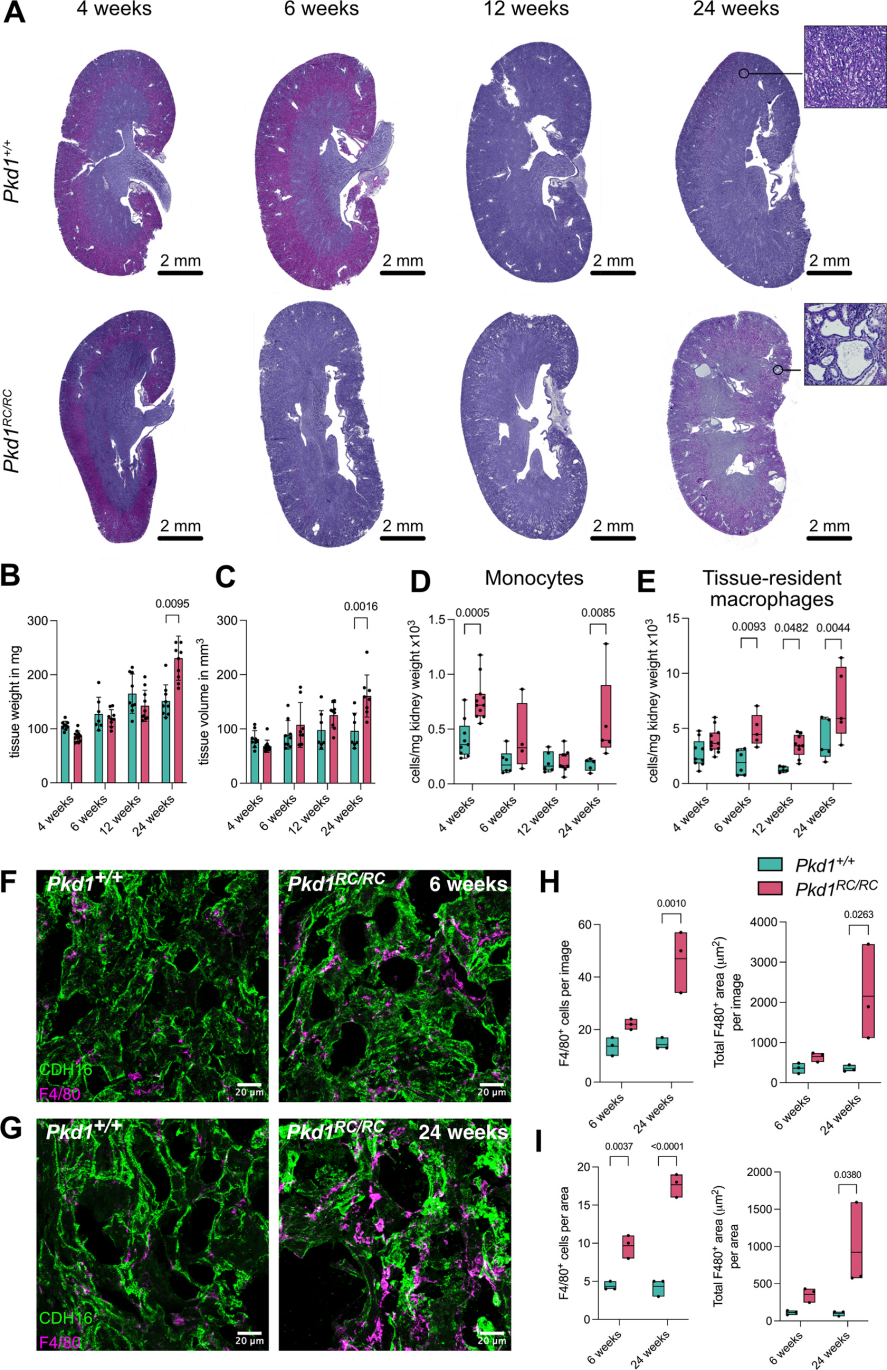
**Characterization of renal macrophages during PKD progression.** (A) Tissue-sections of *Pkd1*^+/+^ (top) or *Pkd1*^RC/RC^ (bottom) kidneys isolated at 4, 6, 12 and 24 weeks of age and stained with H&E. (B) Kidney weight and (C) kidney volume. Data are shown as mean±s.d.; *n* numbers (mice) are indicated in the graph as individual dots (*Pkd1*^*+/+*^ *n*=9, 7, 9 and 9 for 4, 6, 12 and 24 weeks, respectively; *Pkd1*^*RC/RC*^ *n*=11, 9, 9 and 10 for 4, 6, 12 and 24 weeks, respectively). (D,E) Changes in renal monocyte (D) and tissue-resident macrophage populations (E) at 4, 6, 12 and 24 weeks of age. All populations are shown as absolute cell numbers (cells/mg). Data are shown as box plots where the box represents the 25–75th percentiles, and the median is indicated; whiskers show the complete range; *n* numbers (mice), individual dots (*Pkd1*^*+/+*^ *n*=9, 6, 5 and 5 for 4, 6, 12 and 24 weeks, respectively; *Pkd1*^*RC/RC*^ *n*=11, 5, 9 and 5 for 4, 6, 12 and 24 weeks, respectively). (F,G) Representative regions of renal cryosections from 6-week-old (F) and 24-week-old (G) *Pkd1^+/+^* and *Pkd1^RC/RC^* mice, labeled with a CDH16 antibody (green, epithelium) and F4/80 antibody (magenta, macrophages). Shown are maximum intensity projections of four Z-stacks (1 µm each). (H) Quantification of the number of F4/80^+^ cells (left) and total F4/80^+^ area (right) per image. (I) Quantification of the number of F4/80^+^ cells in proximity (left) and the total area covered by F4/80^+^ cells (right) per area defined around the kidney tubule or cyst. Box plots show the mean (and complete range), individual data points represent one mouse, containing 5–9 images per experiment. *P*-values were calculated using a one-way ANOVA with Bonferroni correction.

To characterize macrophages in slowly progressing ADPKD, we used an orthologous model of ADPKD1, the *Pkd1 p.R3277C* mouse (*Pkd1^RC/RC^*) (Arroyo et al., 2021; Hopp et al., 2012) in a C57BL/6JRcc background. *Pkd1^RC/RC^* mice on this background show a slow disease onset and progression with cysts being visible at 24 weeks, but not at 4, 6 or 12 weeks (Fig. 2A). This was also represented in the changes in kidney mass and volume (Fig. 2B,C). We then analyzed the distribution of monocytes and the tissue-resident macrophages during disease progression using flow cytometry (Fig. S2A,B). Monocyte numbers were increased only at 4 and 24 weeks (Fig. 2D), whereas numbers of tissue-resident macrophages were significantly increased from 6 weeks onwards in *Pkd1^RC/RC^* mice compared to *Pkd1^+/+^* mice (Fig. 2E). Of note, neutrophils were only increased at 24 weeks (Fig. S2C), indicating that *Pkd1^RC/RC^* kidneys display an inflammatory environment with both monocytes and neutrophils being recruited to the kidney only when there is a visible cystic phenotype. Taken together, our data indicate that prior to the onset of a pro-inflammatory environment triggered by cyst formation, tissue-resident macrophages in *Pkd1^RC/RC^* mice are already responding to local cues, resulting in their expansion.

### Spatial distribution of renal macrophages

To verify the changes in macrophages in the tissue context during disease progression, we performed fluorescent immunostainings of kidney sections at the different time points of disease progression (Fig. 2F,G). We performed image analysis to determine the abundance of F4/80^+^ macrophages and their proximity to cysts (Fig. S3A–D). The analysis demonstrated that F4/80^+^ macrophages were increased in *Pkd1^RC/RC^* compared *Pkd1^+/+^* mice at 6 and 24 weeks (Fig. 2F–H), and also their abundance in proximity to a cyst was increased at both time points (Fig. 2I). In summary, flow cytometry together with image analysis revealed that tissue-resident macrophages had already increased before disease manifestation in proximity to cystic areas.

### Identification of new macrophage markers during disease progression

**Fig. 3.**
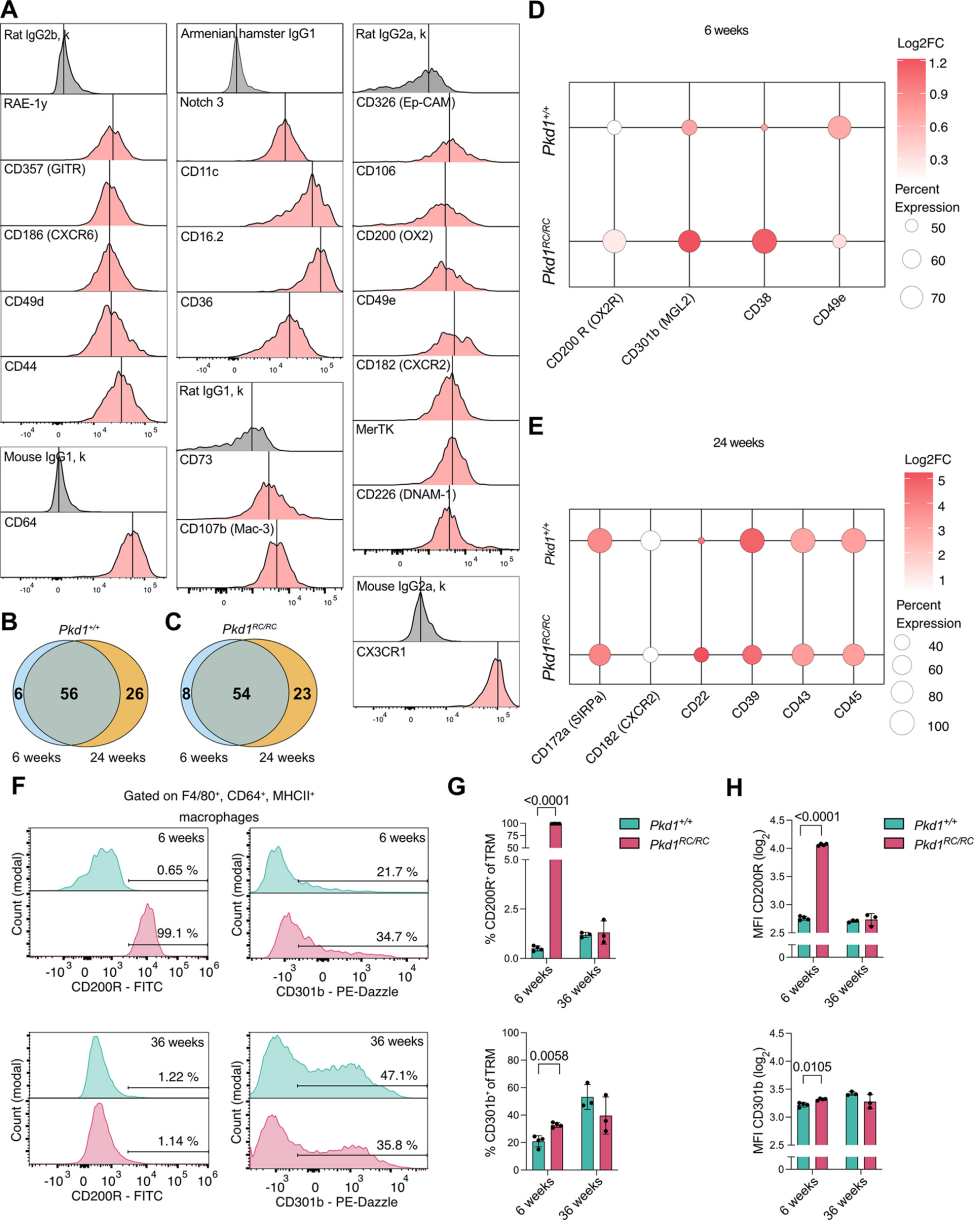
**Changes in macrophage phenotypes before PKD onset and during disease progression.** (A) Histograms of selected top 20% expressed markers on tissue-resident macrophages from 24-week-old *Pkd1^+/+^* mice. Markers are clustered according to the matching isotype control (gray). The vertical black line indicates the mean fluorescence intensity (MFI) of every histogram. (B,C) Venn diagrams showing the overlap of markers identified in *Pkd1^+/+^* (B) and *Pkd1^RC/RC^* (C) mice at 6 and 24 weeks. (D,E) Dot plot showing the most differentially expressed tissue-resident macrophages markers from *Pkd1^+/+^* and *Pkd1^RC/RC^* mice at 6 weeks (D) and 24 weeks of age (E). Dot size represents the percentage of macrophages expressing the respective marker. Log_2_FC values were calculated based on normalized median fluorescence intensity relative to the corresponding isotype control (see Materials and Methods). (F) Histograms of F4/80^+^CD64^+^/MHCII^+^ macrophages expressing CD200R1 or CD301b at 6 or 36 weeks. (G,H) Bar plots showing the percentage (G) and the log_2_ transformed MFI (H) of macrophages from F expressing CD200R1 (top) or CD301b (bottom). Data are shown as mean±s.d. A–E, *n*=3 mice for each genotype (*Pkd1*^*+/+*^ and *Pkd1*^*RC/RC*^) and timepoint (6 and 24 weeks); F–H, *n*=4 (*Pkd1*^*+/+*^, 6 weeks), *n*=5 (*Pkd1*^*RC/RC*^, 6 weeks), *n*=3 (*Pkd1*^*+/+*^, 36 weeks), *n*=3 (*Pkd1*^*RC/RC*^, 36 weeks). *P*-values were calculated using a one-way ANOVA with Bonferroni correction post test.

To further characterize tissue-resident macrophage (Fig. S2A) phenotypes and assess potential changes in surface marker expression during disease progression, we performed a comprehensive flow cytometry screening using the LEGENDScreen™ panel on kidneys from 6- and 24-week-old *Pkd1^+/+^* and *Pkd1^RC/RC^* kidneys (Fig. S4A). We first used the 24-week *Pkd1^+/+^* data to identify known and novel markers that are expressed by at least 90% of all renal macrophages (Fig. S4B). We found MerTK, CD64 (FCGR1), CD11c (ITGAX), CD16.2 (FCGR4), CD44, CD36, CD172a (SIRPA) and CD106 (VCAM1) being expressed, which are broad macrophage markers across tissues (Fig. 3A; Fig. S4B), verifying the functionality of the screening assay. Additionally, we detected CD226 expression, which has been implicated in promoting renal fibrosis (Song et al., 2024), and CD49e (integrin α5, ITGA5), which exhibited heterogenous expression, indicating further diversity within the renal macrophage population (Fig. 3A).

To identify the markers that were up- or down-regulated at different time points of disease development, we compared the data from the flow cytometry screening panel on kidneys from *Pkd1^+/+^* and *Pkd1^RC/RC^* mice at 6 and 24 weeks. In *Pkd1^+/+^* mice, six proteins were only expressed at 6 weeks and 26 were only expressed at 24 weeks, whereas 56 were expressed in common at both stages. In *Pkd1^RC/RC^* mice, eight proteins were only expressed at 6 weeks and 23 were only expressed at 24 weeks, whereas 54 were expressed in common at both stages (Fig. 3B,C; Tables S3, S4). To identify the markers that were specific for the disease, we compared *Pkd1^+/+^* mice and *Pkd1^RC/RC^* mice at 6 and 24 weeks and plotted those where the percentage of cells expressing that marker changed by at least 20% (up- or down-regulated) between genotypes. At 6 weeks, the relative expression and the percentage of cells expressing the marker was distinctively different for CD220R (OX2R; also known as HCRTR2), CD301b (MGL2), CD49e and CD38 between *Pkd1^RC/RC^* and *Pkd1^+/+^* mice (Fig. 3D), presenting suitable candidates for detecting early disease onset. However, at 24 weeks, the difference in relative expression levels between identified markers was not as pronounced between *Pkd1^+/+^* and *Pkd1^RC/RC^* mice and only their cellular abundance slightly changed (Fig. 3E). We verified the differential marker expression at 6 weeks using flow cytometry. Indeed, 99% of tissue-resident macrophages expressed CD200R1 in *Pkd1^RC/RC^* mice at 6 weeks but only 0.65% in *Pkd1^+/+^* mice (Fig. 3F–H). At a later stage, CD200R1 expression was absent in both genotypes, suggesting that its presence on macrophages in *Pkd1^RC/RC^* mice is temporally restricted to early stages. A similar pattern was observed for CD301b, which was highly expressed on tissue-resident macrophages in *Pkd1^RC/RC^* mice at 6 weeks, but minimally in *Pkd1^+/+^* mice (Fig. 3F–H). At a later stage, no difference in CD301b expression was observed between genotypes, indicating that this upregulation is also restricted to early disease stages (Fig. 3F–H).

### Tissue-resident macrophages promote cystogenesis in a 3D model system *in vitro*

**Fig. 4.**
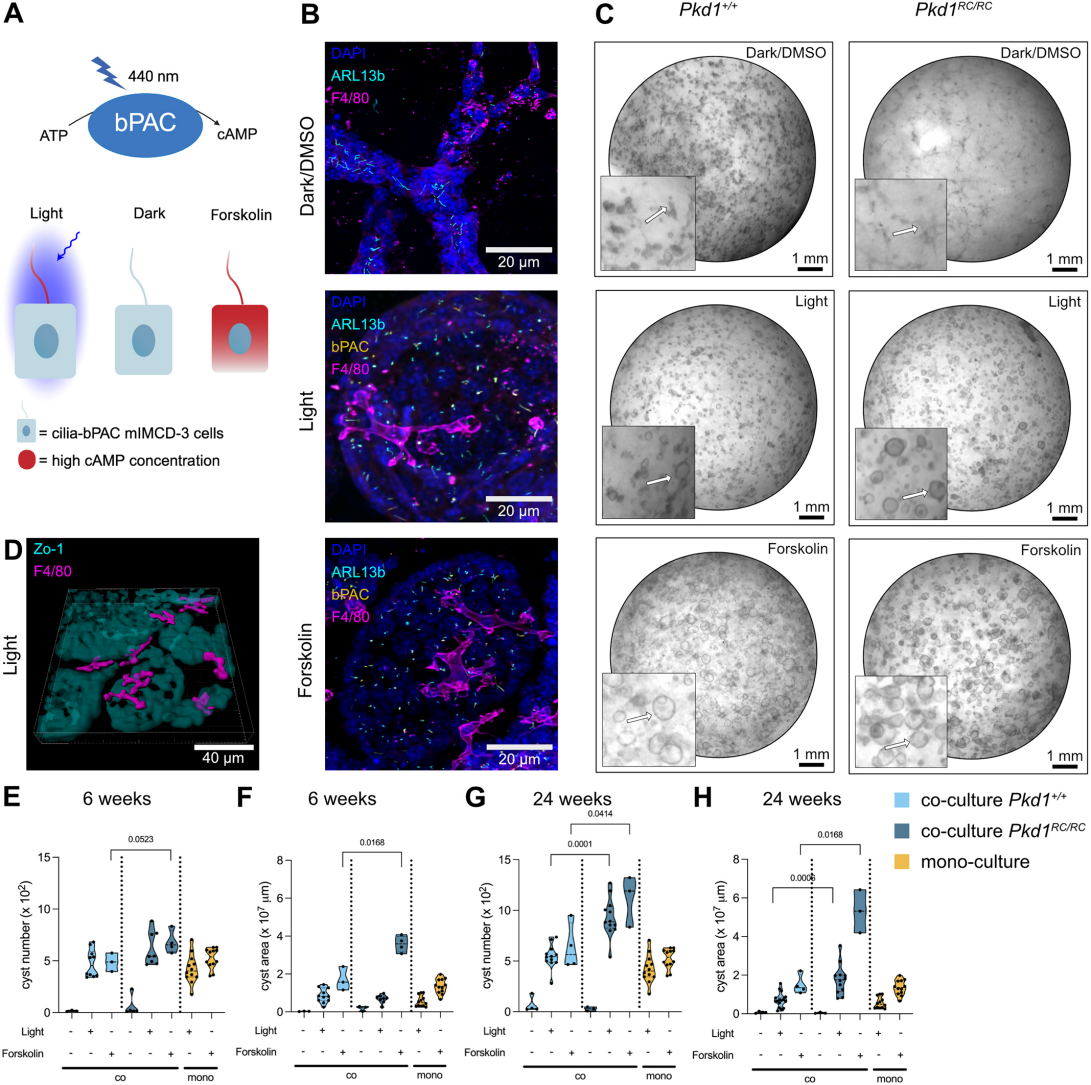
**Tissue-resident macrophages promote cytogenesis in a 3D model system *in vitro*.** (A) Schematic overview optogenetic approach using cilia-bPAC to increase ciliary cAMP levels. Forskolin is used as a control to increase cAMP levels in the whole cell. Created in BioRender by Wachten, D., 2025. https://BioRender.com/mfe4ueq. This figure was sublicensed under CC-BY 4.0 terms. (B) Immunoflorescence stainings of 3D co-cultures using primary renal tissue-resident macrophages of *Pkd1*^RC/RC^ mice (24 weeks). Cells were labeled with the DNA stain DAPI (blue), the ciliary marker ARL13b (cyan) and the macrophage marker F4/80 (magenta). (C) Co-cultures of cilia-bPAC mIMCD-3 cells and primary renal tissue-resident macrophages isolated from *Pkd1*^+/+^ (top) or *Pkd1*^RC/RC^ mice and stimulated with forskolin or light. Arrows in the magnified pictures point towards structures of interest (tubules or cysts). (D) 3D reconstruction of B for cilia-bPAC cells after light stimulation with cysts shown in cyan (ZO-1, also known as TJP1) and macrophages in magenta (F4/80). Cysts are projected 70% translucent. (E–H) Quantification of cystogenesis in mIMCD-3 mono- and co-cultures with renal tissue-resident macrophages isolated from *Pkd1*^+/+^ or *Pkd1*^RC/RC^ mice at 6 weeks (E,F) or 24 weeks (G,H). Quantification was performed for cyst number (E,G) or cyst area (F,H). Individual data points depict one experiment; violin plots show the median and quartiles highlighted by dashed lines; *P*-values were calculated using an unpaired two-tailed Student's *t*-test. For each condition from left to right in E–H: (E) *n*=3, 10, 3, 5, 8, 4, 12, 12; (F) *n*=3, 11, 3, 3, 8, 4, 12, 12; (G) *n*=4, 12, 4, 3, 12, 3, 12, 12; (H) *n*=4, 16, 4, 3, 12, 3, 12, 12.

To investigate the role of tissue-resident macrophages in promoting cystogenesis *in vitro*, we applied a matrix-based mIMCD-3 cell 3D culture system (Elberg et al., 2012). We have previously applied this model to use the photo-activated adenylyl cyclase bPAC in cilia (cilia-bPAC mIMCD-3 cells) and manipulate cAMP signaling with light, demonstrating that ciliary cAMP signaling promotes cystogenesis (Hansen et al., 2022) (Fig. 4A). We added F4/80^+^CD64^+^ renal tissue-resident macrophages, isolated by fluorescence-assisted cell sorting (FACS) from kidneys of *Pkd1^+/+^* mice and *Pkd1^RC/RC^* mice at 6 weeks and 24 weeks (Fig. S5A) to this model to investigate whether the presence of tissue-resident macrophages affects cystogenesis. We first verified the induction of cyst formation by either pharmacologically increasing cAMP levels using forskolin or by specifically increasing the ciliary cAMP concentration using optogenetics (cilia-bPAC) in 3D monocultures and demonstrated that both stimuli promote cystogenesis (Fig. S5B,C). Next, we verified that macrophages and mIMCD-3 cells survive during 3D co-culture (Fig. S5D,E) and demonstrated that they intercalated within the tubular structures and cysts (Fig. 4B,D). When performing co-cultures with tissue-resident macrophages isolated from *Pkd1^+/+^* mice, cyst formation induced either pharmacologically or optogenetically remained unchanged compared to that in monocultures (Fig. 4C,E–H). However, when performing co-cultures with tissue-resident macrophages isolated from 6- or 24-weeks old *Pkd1^RC/RC^* mice, cyst formation was significantly increased compared to what was seen for co-cultures with macrophages from *Pkd1^+/+^* mice and in monocultures (Fig. 4E–H), demonstrating that the remodeling of tissue-resident macrophages has already occurred at an early stage of a mild disease model, which promotes cystogenesis.

Altogether, our study provides new insight into renal macrophage fate and function in ADPKD. Our results reveal that tissue-resident macrophages are increased in *Pkd1^RC/RC^* mice even before renal cysts are formed, and those cells are sufficient to enhance cystogenesis *in vitro*. Furthermore, we identified new macrophage markers showing the potential to detect macrophage remodeling before cysts are formed. Our results are in line with previous results, characterizing macrophage heterogeneity in healthy mice (Yashchenko et al., 2023), demonstrating that HSC-derived monocytes (Ccr2^+^) contribute to the tissue-resident macrophage pool. In the double fate-mapper model, we observed that at 4 weeks of age, ∼25% of F4/80^high^ tissue-resident macrophages are indeed monocyte-derived. This suggests that the PKD-associated microenvironment might promote the recruitment and long-term integration of monocyte-derived macrophages into the resident pool. Our findings further support other studies, which demonstrated accumulation of tissue-resident (CD206^+^) macrophages in a conditional, fast progressing *Pkd2*^−/−^ mouse model, in a Cx3cr1-dependent manner (Li et al., 2023). Using the slow disease onset model in our study allowed us to reveal that the tissue-resident macrophages are already remodeled before cystogenesis, which is sufficient to augment cyst formation *in vitro*. Of note, Cx3cr1 expression was not different between genotypes or ages, and our screening assay did not include Ccr2, whose accumulation is spatially controlled by Cx3cr1 (Yashchenko et al., 2023). The severity of PKD depends on when the disease is induced – deletion of either *Pkd1* or the *Ift88* gene (inducing cilia loss) before the second week of life results in severe phenotype, whereas deletion at a later time point reduces phenotype severity (Davenport et al., 2007; Lehman et al., 2008; Piontek et al., 2007). Our data demonstrate a strong increase in both monocyte and macrophage numbers shortly after birth. Whether the abundance of these cells matters for disease severity needs to be studied further.

As accumulation of tissue-resident macrophages has also been observed in individuals with ADPKD and correlates with renal functional decline (Li et al., 2023), studying the cellular communication between renal epithelial cells and tissue-resident macrophages at disease onset and during progression will pave the way for identifying new targets in treating ADPKD.

## MATERIALS AND METHODS

### Animal studies

All animal experiments were performed in agreement with the German law of animal protection and local institutional animal care committees (Landesamt für Natur, Umwelt und Verbraucherschutz, LANUV). Mice were housed under a circadian light–dark cycle with each at 12 h. Animals were given water and complete diet (ssniff Spezialdiäten) *ad libitum* and breeding was performed as applied for LANUV Az 81-02.04.2019.A428. Mice from both sexes were killed using cervical dislocation. *Pkd1*^RC/RC^ animals were received from Peter C. Harris (PKD Discovery Laboratory, Mayo Clinic, Rochester, MN, USA) and have been previously described (Arroyo et al., 2021; Hopp et al., 2012). To generate the double fate-mapper *Tnfrsf11a^Cre^* (Maeda et al., 2012); *Rosa26^LSL−YFP^* (JAX stock #006148) (Srinivas et al., 2001); *Ms4a3^FlpO^*; *Rosa26^FSF-tdTomato^* (JAX stock #032864) (Daigle et al., 2018), we bred *Tnfrsf11a^Cre/+^; Rosa26^FSF-tdTomato/FSF-tdTomato^* animals with *Ms4a3^FlpO/FlpO^*; *Rosa26^LSL-YFP/LSL-YFP^* animals.

### 3D co-cultures

mIMCD-3 cells (ATCC, # CRL-2123) were maintained in DMEM/F-12 plus GlutaMAX™ Supplement growth medium (Thermo Fisher Scientific, #31331028) containing 10% heat-inactivated fetal bovine serum (Biochrom GmbH) at 37°C and 5% CO_2_. mIMCD-3 cells stably expressing the photoactivated adenylyl cyclase mNphp3(201)-bPAC-mCherry in the cilium (cilia-bPAC) have been previously described (Hansen et al., 2022). mIMCD-3 cells were seeded in a 96-well plate together with primary renal macrophages (for isolation see ‘Flow cytometry and cell sorting’) into a matrix of equal parts collagen I from rat-tail (Gibco, #A10438-01) and Matrigel (Geltrex™) in a 2:1 ratio at a density of 0.6×10^5^ cells/well. After the gels solidified in the incubator for 30 min (37°C, 5% CO_2_), growth medium supplemented with hM-CSF (Miltenyi Biotec) and forskolin (10 µM; Sigma-Aldrich, #F6886) or DMSO as vehicle control. The medium was replaced every 2 days. After 8 days, cells were fixed in 4% paraformaldehyde (Alfa Aesar, Thermo Fisher Scientific, #43368) for 30 min at room temperature and subsequently subjected to microscopy and immunofluorescence staining. Cells had been tested for mycoplasma twice a year and were free from mycoplasma.

### Optogenetic stimulation

For optogenetic stimulation, an RGB LED panel was used (30×30 cm, 18 W, Lichtblick GmbH, Germany, #P1327). Only the blue LED (peak-emission wavelength of 465 nm) was set to a 1 h on, 1 h off cycle with a light intensity of 38.8 μW/cm².

### Flow cytometry and cell sorting

Kidneys were surgically removed from mice directly after killing, minced and digested with 2 mg/ml collagenase II (Life Technologies) and 15 kU/ml DNase I (PanReac, AppliChem) in PBS for 30 min at 37°C under constant agitation. The digestion was quenched by adding FACS buffer [0.5% BSA (Sigma-Aldrich) and 2 mM EDTA (Invitrogen™) in PBS]. The dissociated cells were first passed through a 100 µm, then through a 40 µm strainer (Corning), and subjected to centrifugation for 5 min at 500 ***g*** and 4°C. The supernatant was removed, the pellet was resuspended in red blood-cell lysis buffer (BioLegend), and incubated on ice for 4 min. The reaction was stopped by adding FACS buffer and centrifugation for 5 min at 500 ***g*** and 4°C. The supernatant was removed, and the pellet was resuspended in FACS buffer. The single-cell suspension was subjected to antibody staining for flow cytometric analyses. Antibodies for flow cytometry and cell sorting are described in Table S1.

For flow cytometry, all further steps were performed in a 96-well plate. Isolated cells were incubated with FcR blocking reagent (Miltenyi Biotech) at 4°C for 15 min. After centrifugation (400 ***g***, 2 min, 4°C), cells were washed with FACS buffer and stained with the primary antibody staining mix for 40 min at 4°C. After washing with FACS buffer, the cells were stained with the secondary antibody mix including the viability dye (Hoechst 33258 or Zombie NIR™) and the streptavidin–FITC-coupled antibody for labelling B-, T- and NK-cells (the lineage) or 15 min at 4°C. Cells were washed with FACS buffer and transferred into a 96-deep well plate containing Precision Count Beads™ (BioLegend) for acquisition. Data was acquired on an ID7000 5L Spectral Analyzer (Sony Biotechnology) and analyzed using FlowJo™ version 10.10 (BD Biosciences).

For the novel surface marker analysis, the staining procedure was as described for flow cytometry by including the staining according to the manufacturer's instructions in the LegendSCREEN™ mouse PE kit (BioLegend). Wells were included if they contained more than 200 macrophages, as identified by the gating strategy in Fig. S1. To identify markers expressed by all macrophages at 24 weeks, positive signals were identified by gating using the relevant isotype control, where the positive gate was set to contain a maximum of 5% of positive events. Potential markers of heterogeneity were identified as markers that were expressed in between 25% and 75% of macrophages. Common markers of macrophages were determined to be expressed by at least 90% of macrophages. To identify relative expression to isotype, the median fluorescence intensity of each well was normalized to that of the relevant isotype and then expressed as log_2_ fold change (Log_2_FC). To identify potential disease biomarkers, markers that were upregulated or downregulated by 20% of macrophages were identified, and their relative expression to isotype was calculated as above.

For fluorescent-assisted cell sorting (FACS), the staining procedure was as described for flow cytometry but without adding counting beads. As controls, cells were labeled for each antibody separately and an unstained control was additionally included. Cells were sorted on a MA900 multi-application cell sorter (Sony Biotechnology).

### Immunofluorescence staining

Antibodies for immunofluorescence are described in Table S2. Fixed 3D co-cultures were removed from their wells with a spatula and transferred into a 24-well plate. All following steps were performed at room temperature (RT) if not stated otherwise. Gels were washed twice with PBS and incubated three times in 1% Triton X-100 (Sigma Aldrich, #X100) in PBS for 10 min each. Next, gels were blocked with 1% Triton X-100 and 10% FCS in PBS for 1 h and incubated overnight with the primary antibodies in 1% Triton X-100 and 10% FCS in PBS at 4°C on a rocking shaker. Afterward, gels were washed three times with 1% Triton X-100 and 10% FCS in PBS for 20 min and 1% Triton X-100 in PBS for 10 min each. Gels were incubated with the secondary antibodies and DAPI in 1% Triton X-100 and 10% FCS in PBS for 5 h at RT. After washing three times with Triton X-100 in PBS for 5 min each and once with PBS, cover slips were mounted with Aqua-Poly/Mount (Tebu-Bio, #07918606-20) on a glass slide using custom-build spacers on each side (three layers of adhesive tape) and drops of PBS at the positions where gels were mounted. Object slides were then dried upside down in a hanging position avoiding pressure on the gels for at least 3 h at RT.

The cryosection slides were thawed for 10 min at RT. Circular outlines were defined around each tissue section with a hydrophobic barrier pen. Cryosections were bleached twice with 4.5% H_2_O_2_ and 20 mM NaOH in PBS underneath the beam of an LED lamp (10,000–30,000 lux) for 1 h at RT. After bleaching, the slides were washed three times with PBS/1% Triton X-100 (Sigma Aldrich, #X100) and placed in a humidity chamber. Following, cryosections were blocked with PBS, 1% Triton X-100 and 10% FCS for 3 h at RT and incubated overnight with primary antibodies in PBS, 1% Triton X-100 and 10% FCS at 4°C. Cryosections were washed three times with PBS, 1% Triton X-100 and 10% FCS for 10 min at RT. Next, cryosections were incubated with secondary antibodies and DAPI (1:10,000) in PBS, 1% Triton X-100 and 10% FCS for 4 h at RT. Afterward, cryosections were washed three times with PBS and 1% Triton X-100 for 10 min and washed once with PBS for 10 min. Cryosections were mounted with Aqua-Poly/Mount (Tebu-Bio, #07918606-20) and microscope coverslips. Slides were dried overnight at RT.

### Histology

Renal tissue was fixed overnight in 4% paraformaldehyde (PFA; Alfa Aesar, Thermo Fisher Scientific, #43368) and subsequently processed using the automated Epredia Excelsior AS Tissue Processor (Thermo Fisher Scientific). The tissues were dehydrated and cleared using a clearing agent and xylene (AppliChem) before being incubated in molten paraffin wax (Labomedic). The tissues were then cast into molds along with liquid paraffin and cooled to form solid paraffin blocks containing the embedded tissue. This process utilized the Leica EG1150 H Paraffin Embedding Station and the Leica EG1150 C Cold Plate. Once embedded, the paraffin-embedded renal tissue was sliced into 4 µm sections with a Thermo Scientific HM 355S Automatic Microtome and mounted on Surgipath X-tra Microscope Slides (Leica Biosystems). For histological analysis, the kidney sections were stained with Hematoxylin and Eosin (H&E) using the Leica ST5020 Multistainer, followed by the Leica CV5030 Fully Automated Glass Coverslipper.

For cryosections, renal tissue was fixed in 4% PFA and treated with isopentane (Riedel-de Haen) at −80°C for 1 min. The tissue was then placed in an optimal cutting temperature (OCT) compound (Tissue-Tek OCT, Sakura) on a specimen chuck for freezing. Once embedded in OCT, the renal tissue was sliced into 15 μm thick sections using a Thermo Scientific CryoStar NX50 and mounted on Surgipath X-tra microscope slides (Leica Biosystems).

Embedding, slicing and staining were conducted by the Histology Platform of the Cluster of Excellence ImmunoSensation^2^ at University Hospital Bonn.

### Microscopy and image analysis

Bright-field images of 3D cultures were taken on the Stemi 508 (Zeiss) connected to the Axiocam 105 color. Depicted images are shown in grayscale. Images were analyzed using ImageJ (Rueden et al., 2017). A region-of-interest (ROI) was drawn for each cyst to obtain the area. The cyst area was obtained by summing up the areas of all drawn ROIs. The cyst number represents the number of ROIs per image.

Cryosection immunofluorescence images were analyzed using AdipoQ (Sieckmann et al., 2022). To quantify the number and total area of macrophages surrounding a kidney cyst or tubule, two concentric circular regions of interest (ROIs) with diameters of 25 µm and 50 µm were drawn. The inner circle encompassed the cyst or tubule, whereas the area between the two circles (30 µm wide) represented the region in proximity to the kidney cyst or tubule. The number and total area of macrophages were quantified in the disk area. *Z*-stacks (1 µm step size) of fluorescently stained 3D cultures and kidney cryosections were taken on a confocal microscope at the Microscopy Core Facility of the Medical Faculty at the University of Bonn (Leica Stellaris 8) with 20× and 40× multi-immersion objective with applied glycerol immersion, respectively. Depicted images are shown as maximum intensity projections, which were generated using ImageJ.

Images of histological H&E-stained kidney sections were taken on the AxioScan.Z1 widefield microscope at the Microscopy Core Facility of the Medical Faculty at the University of Bonn.

The 3D reconstruction of the cysts and macrophages in Fig. 4D was generated with Imaris (version 9.1.2.). Cysts and macrophages were visualized using the ‘find surface’ function, with background subtraction (local contrast) and surface detail smoothing of 3 µm for macrophages and 6 µm for cysts.

### Statistical analysis

For plotting graphs and statistical analysis GraphPad Prism 10.4.0 (527) software was used. Statistical analysis and *P*-values are as indicated.

## Supplementary Material

10.1242/joces.263992_sup1 Supplementary information
