# Supplementary material for: Renal tissue-resident macrophages promote cystogenesis in early polycystic kidney disease
Source: J Cell Sci. 2025 Aug 26;138(20):jcs263992. doi: 10.1242/jcs.263992 (PMC12450461; doi:10.1242/jcs.263992)
Supplement: Supplementary information [file joces-138-263992-s1.pdf]

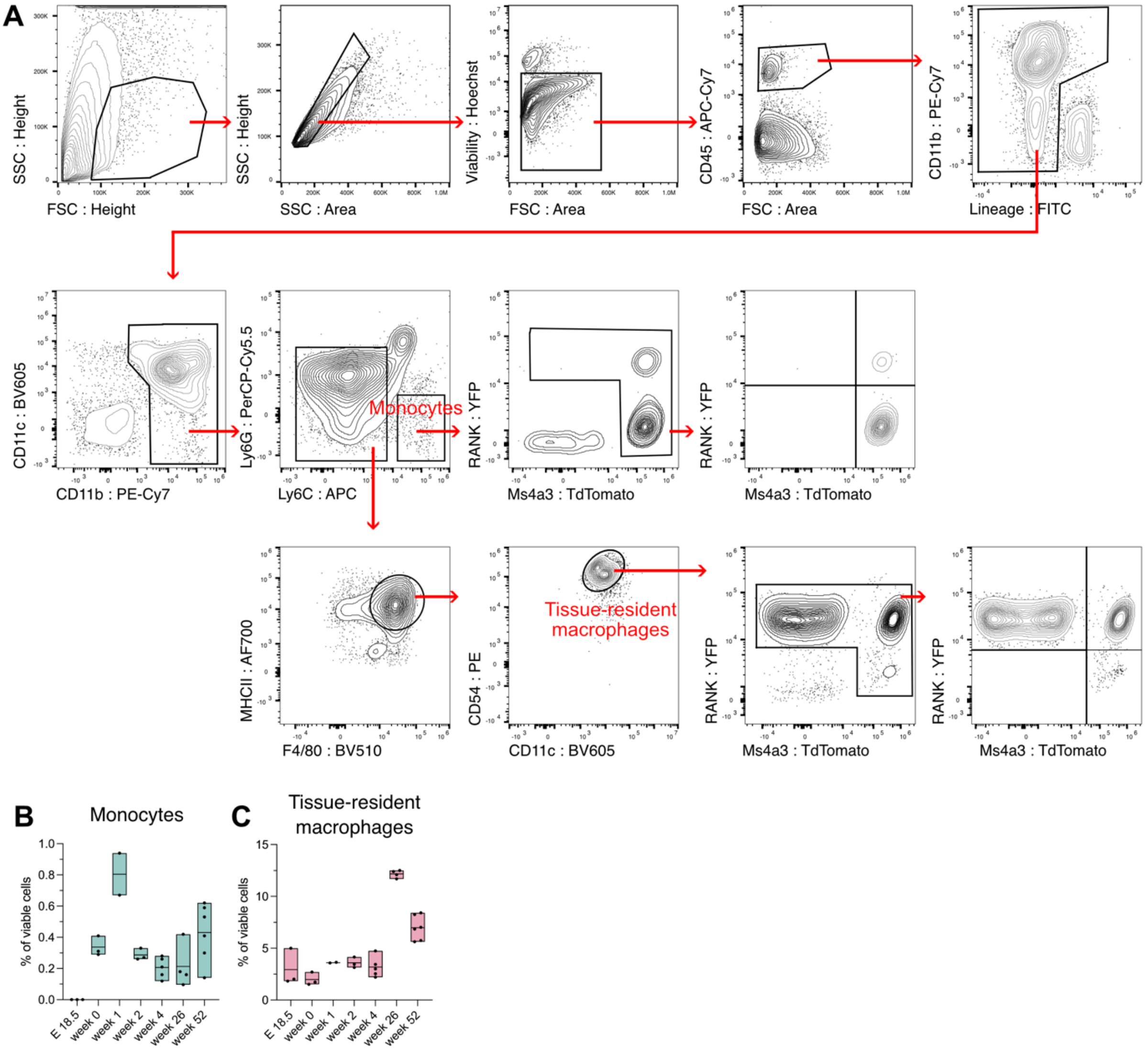

Fig. S1. Characterization of the *double fate-mapper* using flow cytometry. (A) Gating-strategy for the analysis of the *double fate-mapper* mice. Red arrows indicate the gate was selected for the subsequent gating. (B) Monocytes and (C) tissue-resident macrophages in the kidney, plotted as percentage of viable cells.

Journal of Cell Science • Supplementary information

A

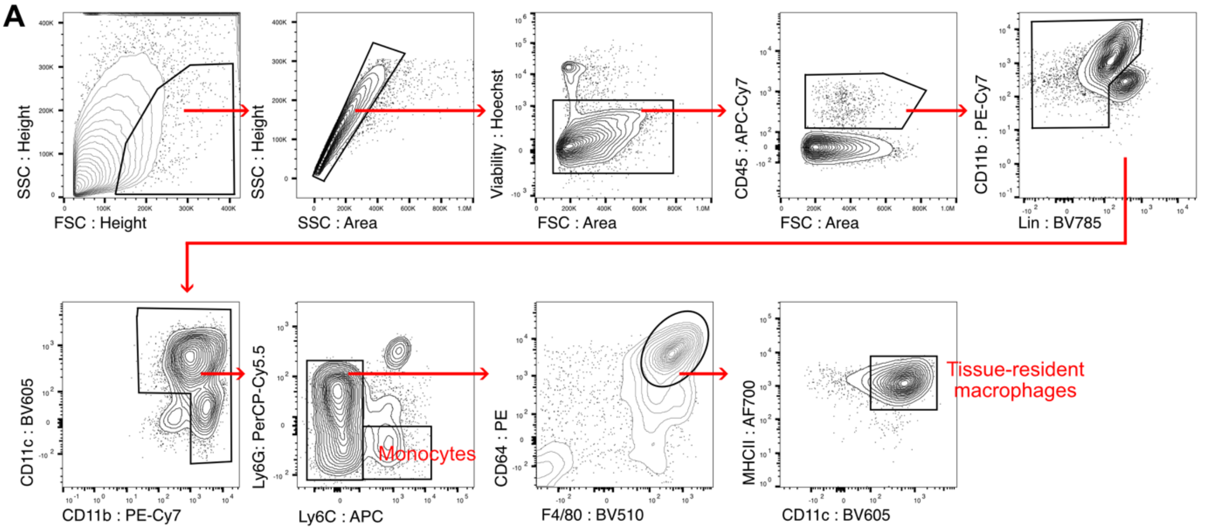

B

Marker	Function
CD45	Pan leukocyte marker
Lineage	exclusion of B-, T- and NK-cells (CD19, TCRb, CD335 (NKp46))
CD11b	Myeloid cell marker, expressed on monocytes, macrophages and neutrophils
CD11c	Marker for monocytes, macrophages and DCs
Ly6C	Monocyte marker (also expressed on neutrophils)
Ly6G	Neutrophil marker
F4/80	Pan macrophage marker
CD64	TRM marker
MHCII	Marker for antigen-presenting cells, highly expressed on TRMs

C

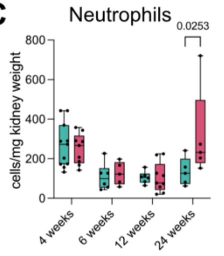

Fig. S2. Characterization of immune cell subsets in a PKD mouse model. (A) Gating-strategy. Red arrows indicate the gate that was selected for the subsequent gating. (B) Overview markers used in (A). (C) Change of the renal neutrophil population from mice either carrying the *Pkd1*$^{+/+}$ (green) or *Pkd1*$^{RC/RC}$ (red) gene over time. Populations are indicated as absolute cell numbers (cells/µl). *P*-values calculated using an unpaired, two-sided Student's *t*-test are indicated.

Journal of Cell Science • Supplementary information

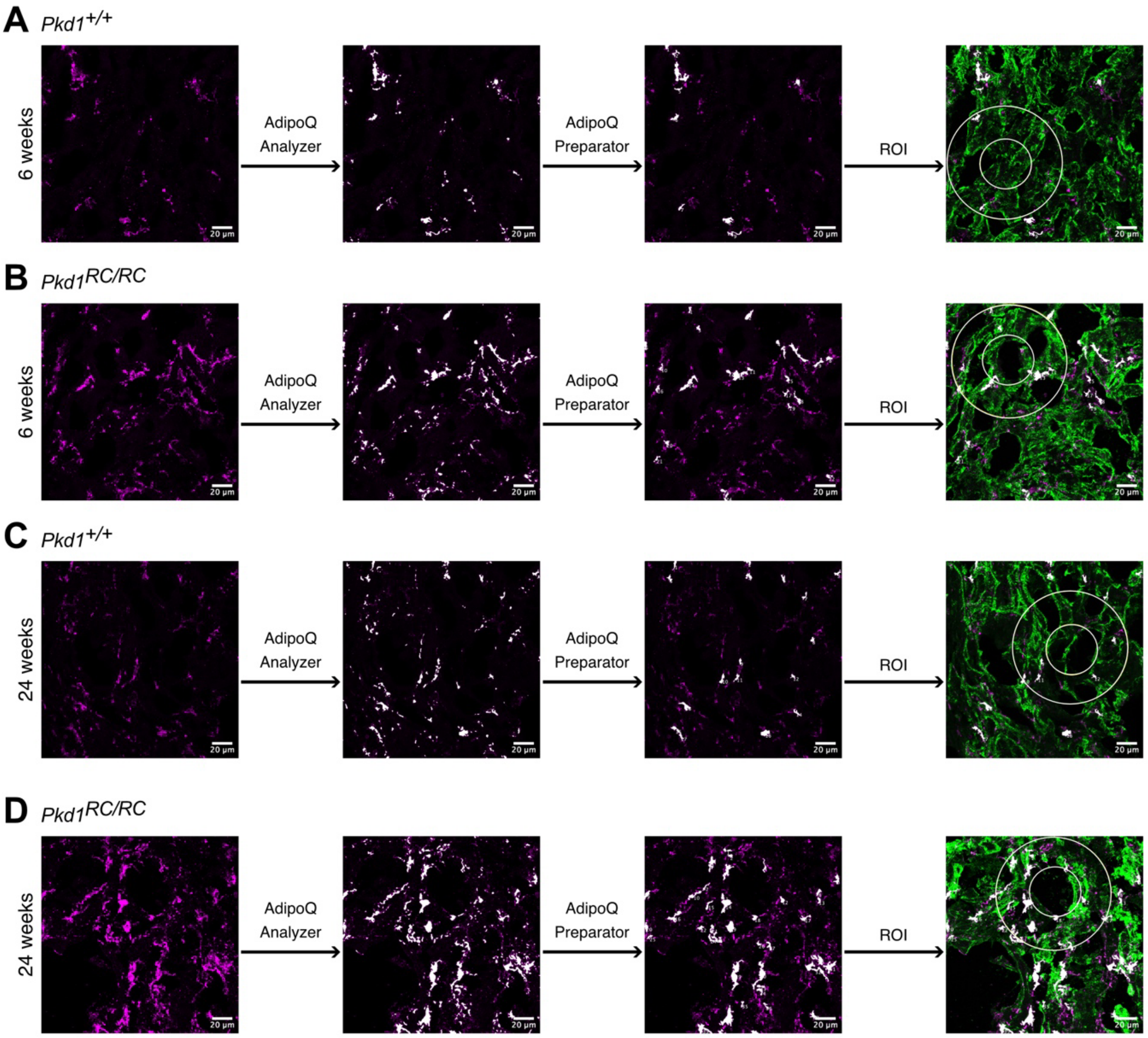

Fig. S3. AdipoQ workflow for analyzing macrophage localization in tissue sections. AdipoQ constitutes a two-step workflow based on the ImageJ plugins, AdipoQ Preparator and AdipoQ Analyzer. Maximum intensity projections of representative regions of renal cryosections from 6-week-old *Pkd1*$^{+/+}$ (A), 6-week-old *Pkd1*$^{RC/RC}$ (B), 24-week-old *Pkd1*$^{+/+}$ (C), and 24-week-old *Pkd1*$^{RC/RC}$ (D) mice, labeled with F4/80 antibody (magenta, macrophages) shown on the left. The AdipoQ Preparator pre-processes the images for optimized segmentation and subsequently segments them into the fore and background, generating a mask that reveals the detected structures (i.e., macrophages, white). The AdipoQ Analyzer quantifies this mask: it counts the structures, determines their size, and determines their area in the images. Followed by quantifying the number and total area of macrophages around one representative kidney cyst or tubule using ROIs. Two concentric circular ROIs (white) were drawn, with diameters of 25 µm and 50 µm, respectively. The inner circle encompassed the kidney cyst or tubule, while the area between the two circles represented the region adjacent to the cyst or tubule. The number of macrophages detected by the mask, as well as their total area, were quantified in the disk region (30 µm wide) between the two circular ROIs. Scale bar: 20 µm.

Journal of Cell Science • Supplementary information

J. Cell Sci.: doi:10.1242/jcs.263992: Supplementary information

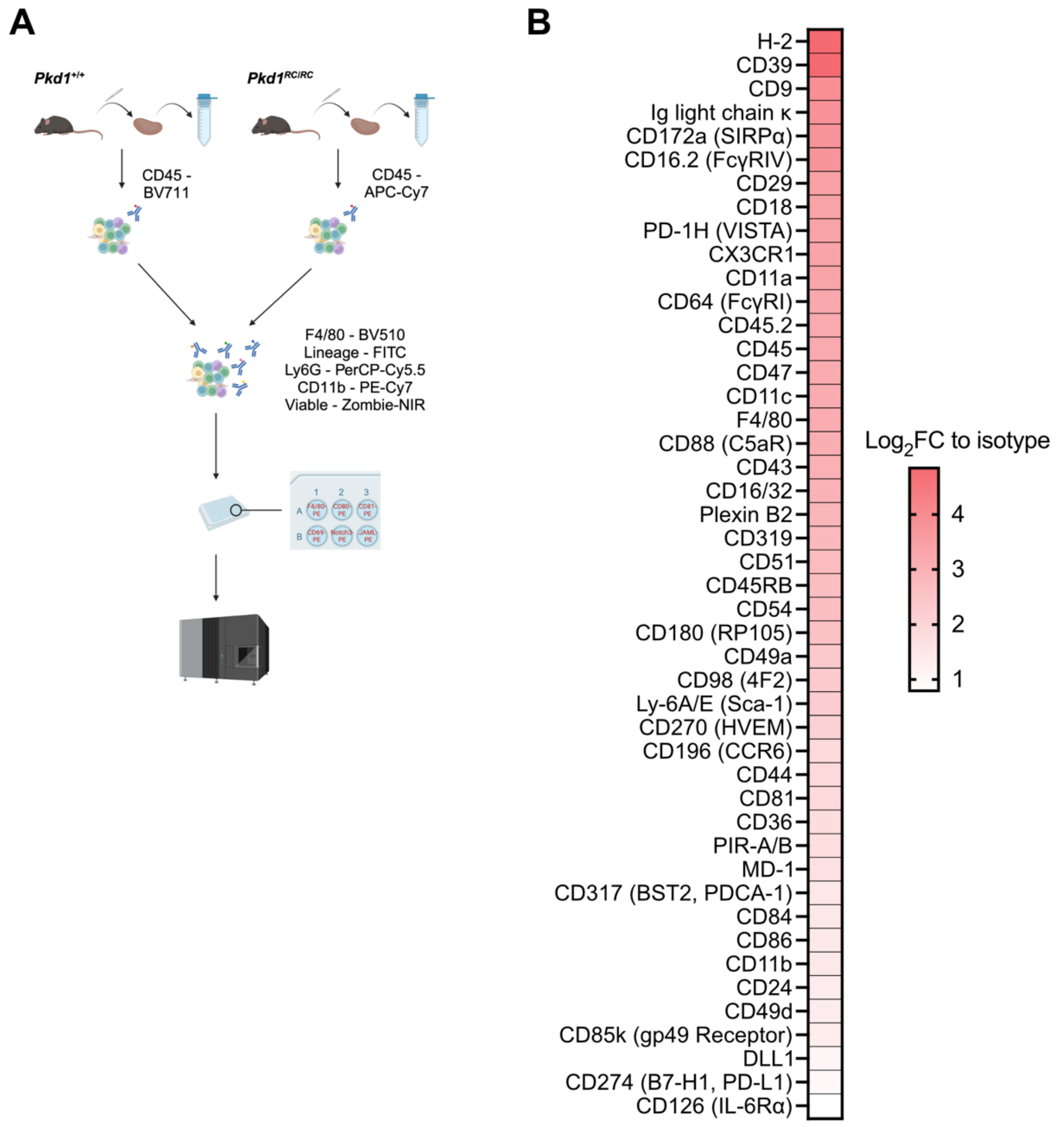

Fig S4. Renal tissue macrophage-niche ontogeny. (A) Schematic overview screening assay. (B) Common kidney macrophage markers (expressed by > 90 % of macrophages), depicted as Log2FC to relative isotype control.

Journal of Cell Science • Supplementary information

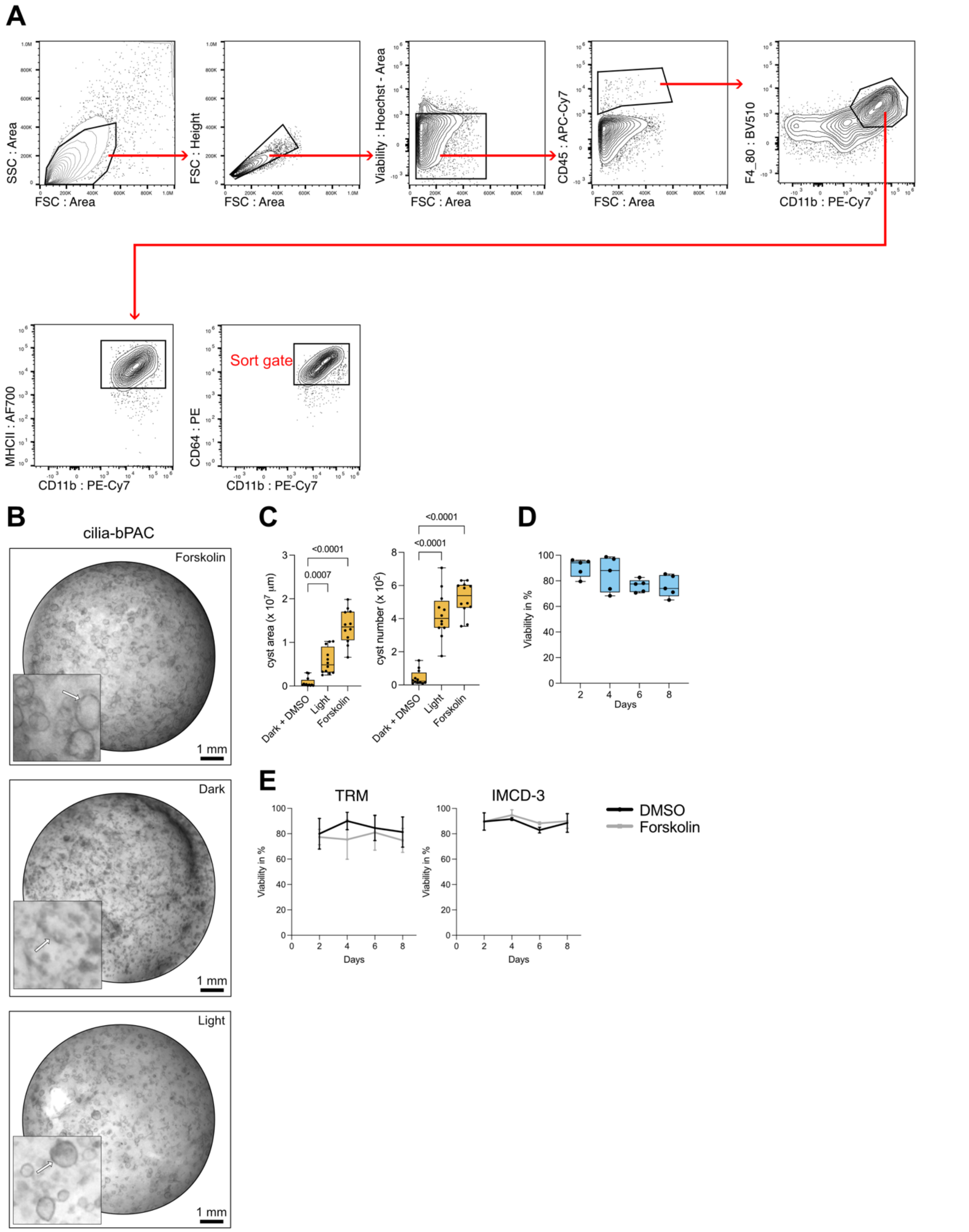

Fig S5. Tissue-resident macrophages promote cystogenesis in a 3D model system *in vitro*. (A) Sorting strategy for primary tissue-resident macrophages from renal tissue. Red arrows indicate which gate was selected for the subsequent gating. (B) Monocultures of cilia-bPAC mIMCD-3 cells cultured in a 3D matrix. Arrows in the zoom-in pictures point towards structures of interest (tubules or cysts). Cells have been stimulated with Forskolin, DMSO (dark), or by light. (C) Quantification of the cyst area and number in 3D monocultures (exemplified in B). (D) Macrophage viability in co-cultures determined by flow cytometry. Each datapoint corresponds to one individual 3D culture. Box plots indicate the 1st and 3rd quartile with the median (min to max); P-values were calculated using a one-way ANOVA.

Journal of Cell Science • Supplementary information

Table S1. Antibodies for flow cytometry.

Panel for cell sorting				
**Reactivity**	**Fluorochrome**	**Dilution**	**Company**	**Cat. No.**
CD45	APC-Cy7	400	Biolegend	103116
CD11b	PE-Cy7	400	Biolegend	101216
F4/80	BV510	100	Biolegend	123135
CD64	PE	200	Biolegend	161004
MHCII	AF700	400	Biolegend	107622
Viability	Hoechst33258	10000	Thermo-Fischer	H1398
**Panel for PKD phenotyping**				
**Reactivity**	**Fluorochrome**	**Dilution**	**Company**	
CD45	APC-Cy7	400	Biolegend	103116
CD11b	PE-Cy7	400	Biolegend	101216
CD11c	BV605	200	Biolegend	117334
F4/80	BV510	100	Biolegend	123135
CD64	PE	200	Biolegend	161004
MHCII	AF700	400	Biolegend	107622
Ly6C	APC	400	Biolegend	128016
Ly6G	PerCP/Cy5.5	200	Biolegend	127616
CD335	Biotin	200	Biolegend	137616
TCR beta	Biotin	200	Biolegend	109204
CD19	Biotin	400	Biolegend	115504
Biotin	FITC	200	Biolegend	405202
Viability	Hoechst33258	10000	Thermo-Fischer	H1398
**Panel for DFM phenotyping**				
**Reactivity**	**Fluorochrome**	**Dilution**	**Company**	
CD45	APC-Cy7	400	Biolegend	103116
CD11b	PE-Cy7	400	Biolegend	101216
CD11c	BV605	200	Biolegend	117334
F4/80	BV510	100	Biolegend	123135
CD64	PE	200	Biolegend	161004
MHCII	AF700	400	Biolegend	107622
Ly6C	APC	400	Biolegend	128016
Ly6G	PerCP/Cy5.5	200	Biolegend	127616
CD335	Biotin	200	Biolegend	137616
TCR beta	Biotin	200	Biolegend	109204
CD19	Biotin	400	Biolegend	115504
Biotin	BV785	200	Biolegend	405249
Viability	Hoechst33258	10000	Thermo-Fischer	H1398

Journal of Cell Science • Supplementary information

Panel for LegendScreen backbone staining				
**Reactivity**	**Fluorochrome**	**Dilution**	**Company**	
CD45	APC-Cy7	400	Biolegend	103116
CD45	BV711	400	Biolegend	103147
CD11b	PE-Cy7	400	Biolegend	101216
F4/80	BV510	100	Biolegend	123135
Ly6G	PerCP/Cy5.5	200	Biolegend	127616
CD335	Biotin	200	Biolegend	137616
TCR beta	Biotin	200	Biolegend	109204
CD19	Biotin	400	Biolegend	115504
Biotin	FITC	200	Biolegend	405202
Viability	Zombie NIR	1000	Biolegend	423106

Table S2. Antibodies for immunofluorescent stainings.

Reactivity	Species	Fluorochrome	Dilution	Company	Cat. No.
ARL13b	Mouse	NA	2000	Abcam	ab136648
ARL13b	Rabbit	NA	500	Proteintech	17711-1-AP
DNA	-	DAPI	10000	Invitrogen	D1306
F4/80	Rat	NA	100/250	Bio-Rad	MCA497GA
Ksp-Cadherin/CDH16	Mouse	NA	250	NeoBiotechnologies	1014-MSM1-P0
Mouse-Ig	Donkey	Alexa Fluor 488	400	Dianova	
Mouse-Ig	Goat	Alexa Fluor 488	400	Invitrogen	A-11029
Rabbi-Ig	Goat	Alexa Fluor 647	150	Jackson ImmunoResearch	111-605-144
Rat-Ig	Donkey	Alexa Fluor 647	150	Dianova	712-605-153

Journal of Cell Science • Supplementary information

Table S3. Common and unique proteins between 6 and 24 weeks in *Pkd1*$^{+/+}$.

**6 weeks *Pkd1*$^{+/+}$**	**24 weeks *Pkd1*$^{+/+}$**
PD-1H (VISTA)	**PIR-A/B**
CD275 (B7-H2, B7-RP1, ICOS Ligand)	**CD172a (SIRPα)**
Ly-6A/E (Sca-1)	**CD319**
CD47	PD-1H (VISTA)
CD98 (4F2)	CD275 (B7-H2, B7-RP1, ICOS Ligand)
**CD14**	Ly-6A/E (Sca-1)
CD107a (LAMP-1)	**CD40**
CD18	CD47
**H-2**	CD98 (4F2)
CD45RB	CD107a (LAMP-1)
CD326 (Ep-CAM)	CD18
CD200 R (OX2R)	**Ly-51**
CD9	**CD71**
CD105	CD45RB
CD366 (Tim-3)	CD326 (Ep-CAM)
CD5	**CD155 (PVR)**
F4/80	CD200 R (OX2R)
CD180 (RP105)	CD9
CD11a	CD105
CD106	CD366 (Tim-3)
CD200 (OX2)	CD5
CD39	F4/80
CD371 (CLEC12A)	CD180 (RP105)
CD63	CD11a
CD49e	**lymphotoxin beta receptor (LTßR)**
CD301a (MGL1)	CD106
CD304 (Neuropilin-1)	**CD115 (CSF-1R)**
MERTK (Mer)	**PDC-TREM**
CD38	CD200 (OX2)
CD301b (MGL2)	**Galectin-9**
CD126 (IL-6Rα)	**MAIR-IV (CLM-5)**
CD86	**CD123**
CD4	CD39
CD54	CD371 (CLEC12A)
CD90.2	CD63
CD49d	CD49e
CD24	CD301a (MGL1)
CD1d (CD1.1, Ly-38)	CD304 (Neuropilin-1)
CD11b	**CD182 (CXCR2)**
CD45	**MAdCAM-1**

Journal of Cell Science • Supplementary information

RAE-1γ	MERTK (Mer)
CD8b	**CD226 (DNAM-1)**
CD44	**LY6K**
CD43	**CD150 (SLAM)**
CD317 (BST2, PDCA-1)	**CD25**
CD132 (common γ chain)	CD38
CD274 (B7-H1, PD-L1)	**CD133**
CD88 (C5aR)	CD301b (MGL2)
MD-1	**CD34**
CD357 (GITR)	**CD154 (CD40L)**
**CD300c/d**	**TIGIT (Vstm3)**
CD186 (CXCR6)	CD126 (IL-6Rα)
CD198 (CCR8)	CD86
IL-23R	**CD37**
**CD184 (CXCR4)**	**CD365 (Tim-1)**
CD2	CD4
CD183 (CXCR3)	CD54
CD16/32	CD90.2
CD279 (PD-1)	CD49d
**CD31 (PECAM-1)**	CD24
Ly-6C	CD1d (CD1.1, Ly-38)
**CD278 (ICOS)**	CD11b
	CD45
	RAE-1γ
	CD8b
	CD44
	CD43
	CD317 (BST2, PDCA-1)
	CD132 (common γ chain)
	CD274 (B7-H1, PD-L1)
	CD88 (C5aR)
	MD-1
	CD357 (GITR)
	CD186 (CXCR6)
	CD198 (CCR8)
	IL-23R
	CD2
	CD183 (CXCR3)
	CD16/32
	CD279 (PD-1)
	Ly-6C
	**GL7 antigen (T and B cell Activation Marker)**

Common proteins in italics, unique proteins in bold.

Table S4. Common and unique proteins between 6 and 24 weeks in *Pkd1*$^{RC/RC}$.

6 weeks *Pkd1*$^{RC/RC}$	24 weeks *Pkd1*$^{RC/RC}$
PD-1H (VISTA)	**PIR-A/B**
CD275 (B7-H2, B7-RP1, ICOS Ligand)	**CD22**
Ly-6A/E (Sca-1)	**CD172a (SIRPα)**
CD47	**CD319**
CD98 (4F2)	PD-1H (VISTA)
**CD14**	CD275 (B7-H2, B7-RP1, ICOS Ligand)
CD107a (LAMP-1)	Ly-6A/E (Sca-1)
CD18	**CD40**
CD71	CD47
**H-2**	CD98 (4F2)
CD45RB	CD107a (LAMP-1)
CD326 (Ep-CAM)	CD18
CD200 R (OX2R)	CD71
CD9	CD45RB
CD105	CD326 (Ep-CAM)
CD366 (Tim-3)	**CD155 (PVR)**
F4/80	CD200 R (OX2R)
CD180 (RP105)	CD9
CD11a	CD105
CD106	CD366 (Tim-3)
CD200 (OX2)	CD5
**CD39**	F4/80
CD371 (CLEC12A)	CD180 (RP105)
CD63	CD11a
CD49e	**lymphotoxin beta receptor (LTßR)**
CD301a (MGL1)	CD106
CD304 (Neuropilin-1)	**CD115 (CSF-1R)**
MERTK (Mer)	CD200 (OX2)
CD38	**MAIR-IV (CLM-5)**
CD301b (MGL2)	CD371 (CLEC12A)
CD126 (IL-6Rα)	CD63
CD86	CD49e
CD4	CD301a (MGL1)
CD54	CD304 (Neuropilin-1)
CD90.2	**CD182 (CXCR2)**
CD49d	**MAdCAM-1**
CD24	MERTK (Mer)
CD1d (CD1.1, Ly-38)	**CD226 (DNAM-1)**
CD11b	**LY6K**
CD45	**CD150 (SLAM)**

RAE-1γ	**CD25**
CD8b	CD38
CD44	**CD133**
CD43	CD301b (MGL2)
CD317 (BST2, PDCA-1)	**CD34**
CD132 (common γ chain)	**CD154 (CD40L)**
CD274 (B7-H1, PD-L1)	**TIGIT (Vstm3)**
**CD88 (C5aR)**	CD126 (IL-6Rα)
MD-1	CD86
CD357 (GITR)	**CD37**
CD186 (CXCR6)	**CD365 (Tim-1)**
CD198 (CCR8)	CD4
IL-23R	CD54
**CD184 (CXCR4)**	CD90.2
CD2	CD49d
CD16/32	CD24
**CD279 (PD-1)**	CD1d (CD1.1, Ly-38)
CD31 (PECAM-1)	CD11b
Ly-6C	CD45
**Podoplanin**	RAE-1γ
**CD278 (ICOS)**	CD8b
	CD44
	CD43
	CD317 (BST2, PDCA-1)
	CD132 (common γ chain)
	CD274 (B7-H1, PD-L1)
	MD-1
	CD357 (GITR)
	**CD300c/d**
	CD186 (CXCR6)
	CD198 (CCR8)
	IL-23R
	CD2
	CD16/32
	CD31 (PECAM-1)
	Ly-6C

Common proteins in italics, unique proteins in bold.

Journal of Cell Science • Supplementary information

Table S5. Mouse genders.

	Timepoint		Females	Males
**Figure 1 A, B, E, F Figure S1 B, C**	E18.5		1	2
	week 0		5	5
	week 1		0	2
	week 2		2	1
	week 4		2	3
	week 26		4	0
	week 52		0	5
**Figure 2 B, C**	**Timepoint**	**Genotype**	**Females**	**Males**
	4 weeks	*Pkd1*$^{+/+}$	4	5
		Pkd1$^{RC/RC}$	7	4
	6 weeks	*Pkd1*$^{+/+}$	4	3
		Pkd1$^{RC/RC}$	6	3
	12 weeks	*Pkd1*$^{+/+}$	7	2
		Pkd1$^{RC/RC}$	5	4
	24 weeks	*Pkd1*$^{+/+}$	7	2
		Pkd1$^{RC/RC}$	5	5
**Figure 2 D, E**	**Timepoint**	**Genotype**	**Females**	**Males**
**Figure S2 B**	4 weeks	*Pkd1*$^{+/+}$	4	5
		Pkd1$^{RC/RC}$	7	4
	6 weeks	*Pkd1*$^{+/+}$	4	2
		Pkd1$^{RC/RC}$	2	3
	12 weeks	*Pkd1*$^{+/+}$	3	2
		Pkd1$^{RC/RC}$	5	4
	24 weeks	*Pkd1*$^{+/+}$	4	1
		Pkd1$^{RC/RC}$	2	3
**Figure 3 A, B, C, D, E**	**Timepoint**	**Genotype**	**Females**	**Males**
	6 weeks	*Pkd1*$^{+/+}$	3	0
		Pkd1$^{RC/RC}$	3	0
	24 weeks	*Pkd1*$^{+/+}$	3	0
		Pkd1$^{RC/RC}$	2	1
**Figure 3 F, G**	**Timepoint**	**Genotype**	**Females**	**Males**
	6 weeks	*Pkd1*$^{+/+}$	4	0
		Pkd1$^{RC/RC}$	0	5
	36 weeks	*Pkd1*$^{+/+}$	3	0
		Pkd1$^{RC/RC}$	2	1
